# Common psychiatric symptoms among public school teachers in Palmas, Tocantins, Brazil. An observational cross-sectional study: Sintomas psiquiátricos comuns em professores das escolas públicas de Palmas, Tocantins, Brasil. Um estudo observacional transversal

**DOI:** 10.1590/1516-3180.2014.8242810

**Published:** 2015-07-03

**Authors:** Leonardo Baldaçara, Álvaro Ferreira Silva, José Gerley Díaz Castro, Gessi de Carvalho Araújo Santos

**Affiliations:** I PhD. Professor, Medicine Program and Master´s Health Science Program, Universidade Federal do Tocantins (UFT), Palmas, Tocantins, Brazil; II MSc. Researcher, Master's Health Science Program, Universidade Federal do Tocantins (UFT), Palmas, Tocantins, Brazil; III PhD. Professor, Master's Health Science Program, Universidade Federal do Tocantins (UFT), Palmas, Tocantins, Brazil

**Keywords:** Teaching, Mental health, Work, Prevalence, Faculty, Ensino, Saúde mental, Trabalho, Prevalência, Docentes

## Abstract

**CONTEXT AND OBJECTIVE:**

Teachers are at great risk of physical and mental stress due to material or psychological difficulties associated with their work. This study aimed to assess the prevalence of common psychiatric symptoms measured on the Self-Reporting Questionnaire (SRQ-20) scale that would suggest a diagnosis of psychiatric disorders among public school teachers in Palmas, Tocantins, Brazil, in 2012.

**DESIGN AND SETTING:**

Observational cross-sectional study in Palmas, Tocantins, Brazil.

**METHOD:**

We assessed 110 municipal teachers in the city of Palmas, Tocantins, Brazil. They were selected randomly from a list of employees of the Municipal Education Department of Palmas. All of them answered the SRQ-20 questionnaire after giving their consent.

**RESULTS:**

Between the years 2008 and 2011, 24 cases of absence from work due to mental disorders were found. We excluded one case and 109 teachers answered the SRQ-20questionnaire. Out of the 109 teachers assessed, 54 had ≥ 7 points on the SRQ-20 scale. This finding suggests that 49.5% of the teachers had symptoms that were sufficient to consider a diagnosis of mental disorder, with the need for treatment.

**CONCLUSION:**

Our study found that the prevalence of mental disorders among teachers is as high as seen in the literature. Our results suggest that recognition of mental disorders is low and that the current statistics fail to reach the occupational health sector.

## Abstract (pt)

**CONTEXTO E OBJETIVO:**

Professores estão sob grande risco de estresse físico e mental devido às dificuldades materiais e psicológicas do seu trabalho. Este estudo visa avaliar a prevalência dos sintomas psiquiátricos comuns medidos pela escala Self-Reporting Questionnaire (SRQ-20), que sugere presença do diagnóstico de transtorno psiquiátrico, em professores de escolas públicas de Palmas, Tocantins, Brasil, no ano de 2012.

**TIPO DE ESTUDO E LOCAL:**

Estudo observacional transversal em Palmas, Tocantins, Brasil.

**MÉTODO:**

Avaliamos 110 professores municipais na cidade de Palmas, Tocantins, Brasil. Eles foram randomicamente selecionados por uma lista de funcionários da Secretaria Municipal de Educação de Palmas. Todos responderam ao questionário SRQ-20 depois darem consentimento.

**RESULTADOS:**

Entre os anos de 2008 a 2011, foram observados 24 casos de abstenção ao trabalho por doenças mentais. Nós excluímos um caso e 109 professores responderam ao questionário SRQ-20. Dos 109 professores avaliados, 54 tinham ≥ 7 pontos no SRQ-20. Esse dado sugere que 49,5% dos professores têm sintomas suficientes para se considerar o diagnóstico de transtorno mental e a necessidade de tratamento.

**CONCLUSÃO:**

Nosso estudo observou que a prevalência de doenças mentais em professores é tão alta como encontrada na literatura. Nossos resultados sugerem que o reconhecimento dos transtornos mentais é baixo e que as estatísticas atuais falham em alcançar o setor de saúde ocupacional.

## INTRODUCTION

Research worldwide has shown that school teachers are at great risk of physical and mental stress due to material or psychological difficulties associated with their work.^1^ Studies conducted in other countries have demonstrated that there is a direct relationship between higher levels of stressors at work and presence of fatigue, sleep disturbances, depressive symptoms and drug consumption.^2^^-^ 5 In Brazil, changes to how the job of teaching is organized, the advent of new requirements and competencies that are changing teaching activity, but without the means to provide compatibility, has created work overload. The factors mentioned above may be a source of complaints, mental disorders and absence from work.^1^ The United Nations Educational, Scientific and Cultural Organization (UNESCO) has reported a progressive increase in violence in schools throughout the world and has interpreted violence in schools as one of the main causes of teachers' distress.^6^

## OBJECTIVE

This study aimed to assess the prevalence of psychiatric symptoms measured by means of the Self-Reporting Questionnaire (SRQ-20) scale, that would suggest a diagnosis of psychiatric disorders among public school teachers in Palmas, Tocantins, Brazil, in 2012.

## METHODS

### Subjects

We assessed teachers working at public schools in the city of Palmas, Tocantins, Brazil. They were selected by randomization in groups from a list of employees of the Municipal Education Department of Palmas. Each group had five workers, i.e., five were called to the survey, five ignored, five were called and so on until the number of 110 subjects was reached (randomization in blocks). They answered the SRQ-207-9 questionnaire after giving their consent.

The inclusion criteria were that the subjects needed to be more than 20 years of age and to have been public teachers for at least a year. The exclusion criteria were the presence of severe physical disease that compromised their mental capacity and working for less than a year as a public teacher. One subject refused to participate after having answered the questionnaire. 

The sample size was calculated in accordance with the National Statistical Service,^10^ using a 95% confidence level, population size of 2,300, proportion of psychiatric disorders of 50% (based on previous studies) and relative standard error of 10%. The minimum sample size was 96 subjects, but we used 110 to avoid losses.

### Diagnostic and symptom assessment

We used the Self-Reporting Questionnaire (SRQ),7-9 which was developed by the World Health Organization. The SRQ assesses factors relating to mental health belonging to different instruments for assessment of mental disorders, such as the General Health Questionnaire (GHQ-60), which is an instrument containing 60 items; a reduced version of this called the Present State Examination (PSE); the Post Graduate Institute Health Questionnaire N 2 (PGI), which was developed in India; and the Patient Symptom Self Report (PASSR), which is an instrument developed in Colombia. This is a self-reporting questionnaire and the optimum cutoff value for the SRQ-20 was 7.

### Statistical analysis

We compared teachers with 7 points or more (≥ 7 points) from the SRQ-20 with teachers with less than 7 points (< 7 points). Age was a continuous variable and was presented as the mean and standard deviation. The two groups were compared by means of the Mann-Whitney test. The other variables (gender, age, marital status, physical activities, workload, and working in a private school) were presented as numbers and proportions. The two groups were compared by means of the chi-square test. The significance level was set at P < 0.10.

## RESULTS

One teacher was excluded from this study because he refused to complete the examination for personal reasons. Among the 109 teachers assessed, 54 had ≥ 7 points on the SRQ-20 scale. This finding suggests that 49.5% of the teachers had symptoms that were sufficient to consider a diagnosis of mental disorder, with the need for treatment.

We compared teachers with ≥ 7 points with teachers with < 7 points and observed that there were no differences in relation to gender, age, marital status, workload and working exclusively in a public school or not. In addition, the proportion of the teachers who practiced physical activity was lower in the group with ≥ 7 points (n = 24, 44.4%) on the SRQ-20 scale, than in the group with < 7 points (n = 32; 58.2%; P = 0.08) (Table 1). Teachers who did not practice physical activity and had ≥ 7 points had an odds ratio of 1.74.

**Table 1.** Main variables assessed among public teachers in Palmas, Tocantins, Brazil, who answered the SRQ-20 questionnaire

Variables	7 or more points	Less than 7 points	Odds ratio	Z/χ^2^	P
Age (mean, years)	34.9 ± 5.1	38.2 ± 6.1	-	1.42	0.33
Gender					
Male	10 (18.5%)	15 (27.3%)	0.60	1.18	0.14
Female	44 (81.5%)	40 (72.3%)	1.65		
Marital status					
Married	38 (70.4%)	33 (60.0%)	1.58	1.29	0.13
Single	16 (29.6%)	22 (40.0%)	0.63		
Physical activity					
Yes	24 (44.4%)	32 (58.2%)	0.57	2.05	0.08
No	30 (55.6%)	23 (41.8%)	1.74		
Workload					
40 hours per week	18 (33.3%)	21 (38.2%)	0.81	0.15	0.35
More than 40 hours per week	36 (66.7%)	34 (61.8%)	1.23		
Income					
Sufficient	7 (13.0%)	11 (20.0%)	0.60	0.98	0.17
Not sufficient	47 (87.0%)	44 (80.0%)	1.68		
Also working at private school					
Yes	47 (87.0%)	47 (85.5%)	1.14	0.25	0.32
No	7 (13.0%)	8 (14.5%)	0.87		

SRQ-20 = Self-Reporting Questionnaire; Z = Z-score; χ2= Chi-square.

## DISCUSSION

The Reference Center for Occupational Health (Centro de Referência em Saúde do Trabalhador, CEREST) in Palmas, Tocantins, assessed the prevalence of mental disorders among teachers in municipal schools between the years 2008 and 2011 and observed 24 cases of absence from work due to mental disorders. Since there were 2,300 municipal teachers in the city of Palmas, the prevalence was 1% over four years. The prevalence was one case in 2008, one case in 2009, 11 cases in 2010 and 11 cases in 2011. Although there was an increase in the number of absences due to mental disorders, this number was lower than expected. Therefore, the SRQ-20 questionnaire was administered in order to assess the prevalence of significant psychiatric symptoms and the likelihood of mental illness among 110 municipal teachers in Palmas, Tocantins.

In Australia, Tuettemann found that the prevalence of mental disorders among high school teachers was 44.6%.^11^ Another study conducted in Hong Kong recently also showed that the teaching profession is highly stressful.^2^ About a third of the teachers surveyed showed signs of stress and burnout among their major health problems.^2^ In Brazil, the prevalence of common mental disorders among teachers in Belo Horizonte was 50.3%.^1^ The frequency of psychiatric disorders found among teachers surveyed in Vitória da Conquista, Bahia, Brazil, was twice the frequency in the general population, with no significant difference between men and women. The presence of disorders was related to working conditions (physical and organizational), and the presence of violence.^12^

Some risk factors found in previous studies investigating psychiatric disorders among teachers included: behavior relating to health and morbidity, difficulty in integrating at work, heavy workload; violence at school, perception of work, resources available for work, and physical environment of the school.^1^^,^ 2 ^,^ 13 Reports of violence in schools are made very frequently among teachers, and all forms of aggression committed by students, students' parents, staff or colleagues at work, or people outside of the school have been strongly associated with mental disorders.^1^

We found that the frequency of doing physical activities was lower among subjects with ≥ 7 points on the SRQ-20 scale, and that the teachers who did not practice exercises presented a 1.74 times greater chance of having a mental disorder. Some studies have shown that exercise and physical activity can prevent or delay the onset of different mental disorders, and that they have therapeutic benefits when used as the sole or adjunct treatment for mental disorders.^14^^-^ 16 Therefore, these data show a possibility for preventive action that could be taken.

## CONCLUSION

Our study found that the prevalence of mental disorders among teachers is as high as seen in the literature. Our results suggest that recognition of such disorders is low and that the current statistics fail to reach the occupational health sector. Most of these teachers are probably not getting appropriate care. Educational and preventive measures are necessary, since the welfare of these professionals is reflected in the quality of learning that young people receive. Future research should focus on the impact of applying preventive programs.
